# Diagnostic challenges and prognostic implications of extranodal extension in head and neck cancer: a state of the art review and gap analysis

**DOI:** 10.3389/fonc.2023.1263347

**Published:** 2023-09-20

**Authors:** Christina E. Henson, Ahmad K. Abou-Foul, Daniel J. Morton, Lachlan McDowell, Sujith Baliga, James Bates, Anna Lee, Pierluigi Bonomo, Petr Szturz, Paul Nankivell, Shao Hui Huang, William M. Lydiatt, Brian O’Sullivan, Hisham Mehanna

**Affiliations:** ^1^ Department of Radiation Oncology and Stephenson Cancer Center, University of Oklahoma Health Sciences Center, Oklahoma City, OK, United States; ^2^ Institute of Head and Neck Studies and Education, School of Cancer Sciences, University of Birmingham, Birmingham, United Kingdom; ^3^ Department of Pediatrics and Stephenson Cancer Center, University of Oklahoma Health Sciences Center, Oklahoma City, OK, United States; ^4^ Department of Radiation Oncology, Peter MacCallum Cancer Centre, Melbourne, VIC, Australia; ^5^ Department of Radiation Oncology, Ohio State University Wexner Medical Center, Columbus, OH, United States; ^6^ Department of Radiation Oncology, Winship Cancer Institute of Emory University, Atlanta, GA, United States; ^7^ Department of Radiation Oncology, University of Texas MD Anderson Cancer Center, Houston, TX, United States; ^8^ Department of Radiation Oncology, Azienda Ospedaliero-Universitaria Careggi, Florence, Italy; ^9^ Department of Oncology, University of Lausanne and Lausanne University Hospital, Lausanne, Switzerland; ^10^ Department of Radiation Oncology, Princess Margaret Cancer Centre, University of Toronto, Toronto, ON, Canada; ^11^ Department of Surgery, Creighton University, and Nebraska Methodist Health System, Omaha, NE, United States

**Keywords:** extranodal extension, head and neck cancer, locally advanced head and neck cancer, head and neck pathology, head and neck squamous cell carcinoma

## Abstract

Extranodal extension (ENE) is a pattern of cancer growth from within the lymph node (LN) outward into perinodal tissues, critically defined by disruption and penetration of the tumor through the entire thickness of the LN capsule. The presence of ENE is often associated with an aggressive cancer phenotype in various malignancies including head and neck squamous cell carcinoma (HNSCC). In HNSCC, ENE is associated with increased risk of distant metastasis and lower rates of locoregional control. ENE detected on histopathology (pathologic ENE; pENE) is now incorporated as a risk-stratification factor in human papillomavirus (HPV)-negative HNSCC in the eighth edition of the American Joint Committee on Cancer (AJCC) and the Union for International Cancer Control (UICC) TNM classification. Although ENE was first described almost a century ago, several issues remain unresolved, including lack of consensus on definitions, terminology, and widely accepted assessment criteria and grading systems for both pENE and ENE detected on radiological imaging (imaging-detected ENE; iENE). Moreover, there is conflicting data on the prognostic significance of iENE and pENE, particularly in the context of HPV-associated HNSCC. Herein, we review the existing literature on ENE in HNSCC, highlighting areas of controversy and identifying critical gaps requiring concerted research efforts.

## 1 Introduction

Extranodal extension (ENE) describes the phenomenon of cancer growth from within the lymph node (LN) outward into the perinodal tissues. The critical event is the disruption and penetration of the tumor through the entire thickness of the LN capsule, which normally acts as a barrier impeding tumor extension and is central to the diagnosis and classification of ENE.

ENE was first reported in 1930 in a retrospective analysis of autopsy material of 20 patients with head and neck cancer. The Australian pathologist Rupert Willis described tumor extension beyond the LN into adjacent structures, including soft tissue and bone (1). Almost three decades later, the negative prognostic impact of ENE was demonstrated in breast cancer, followed by similar findings in head and neck squamous cell carcinoma (HNSCC) ten years later (2).

The underlying pathobiology of ENE remains unclear. However, the presence of ENE is often associated with an aggressive cancer phenotype in HNSCC (3) and other tumour types (4). In HNSCC, ENE is associated with an increased risk of distant metastasis and lower rates of locoregional control (5). Since the publication of the two seminal adjuvant therapy trials in 2004 (6–8), ENE detected on histopathology (pathologic ENE; pENE) has also been considered a high risk feature and an indication for treatment intensification by adding cisplatin to radiotherapy (RT) after surgery, albeit at the cost of higher overall toxicity. Consequently, the presence of pENE has now been incorporated as a risk-stratification factor in Human papillomavirus (HPV) negative HNSCC in the latest (8^th^) edition of the American Joint Committee on Cancer (AJCC) and the Union for International Cancer Control (UICC) TNM Staging Manual (9).

Although more than ninety years have passed since the first description of ENE, several issues remain unresolved. These include the lack of consensus on definitions, terminology, and widely accepted assessment criteria and classification systems for both pENE and ENE detected on pre-treatment imaging (imaging-detected ENE; iENE). Moreover, there is no agreement on the prognostic significance of iENE and pENE, particularly in the context of HPV-associated HNSCC. Here, we review the existing literature on ENE, highlighting areas of controversy and identifying critical gaps that need further research.

## 2 Definition and classification of ENE

### 2.1 Pathologic ENE

**Figure 1:**
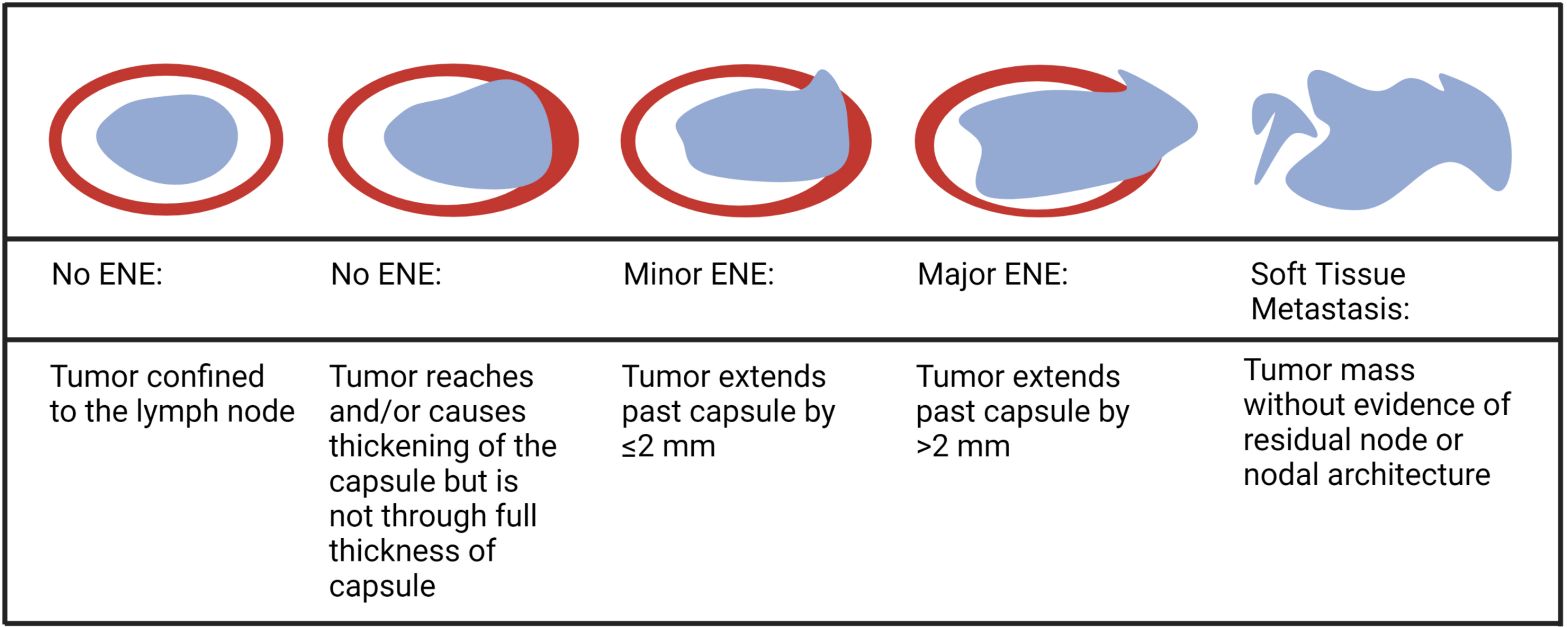
Depiction of various extents of lymph node involvement with tumor (Created in Biorender.com).

pENE is commonly defined as extension of tumor cells outside the LN capsule into the perinodal soft tissue on histopathologic examination (10). It is often subcategorized as either microscopic/minor (≤2 mm in extent) or macroscopic/major (>2 mm in extent) or as a soft tissue metastasis (STM) (**Figure 1**), as recommended by the AJCC for documentation purposes (9). The prognostic significance of the 2 mm extension threshold remains contentious, especially in the context of HPV-associated HNSCC (11–16).

Determining pENE can be complicated by the presence of an incomplete nodal capsule. This can occur as a result of sample preparation, or because the capsule has thinned and is difficult to identify, which is especially common at the hilum of the lymph node (17). When the capsule is deficient, pathologists sometimes elect to reconstruct it virtually based on the remaining evident portions, which can introduce heterogeneity and impact the reproducibility of results (12). Another clinical conundrum may occur where there is continuity of a primary tumor and an adjacent lymph node. Some pathologists may elect to diagnose ENE in this case, as it cannot be definitely excluded, while other pathologists may restrict the diagnosis of pENE to those cases where there is evidence of remnant capsule that is discontinuous with the primary tumor (10). A further challenge arises when grossly confluent LNs (also known as matted or coalescent nodes) are present. In this case, additional sections are recommended to exclude ENE, as confluent LNs may simply represent closely aggregated LNs with thickened capsules without actual microscopic evidence of ENE (10). Despite the aforementioned limitations, histopathology remains the gold standard for determining the presence of ENE, and in most cases, it can be determined and categorized as macroscopic (major) or microscopic (minor) (5).

### 2.2 ENE on imaging

**Figure 2:**
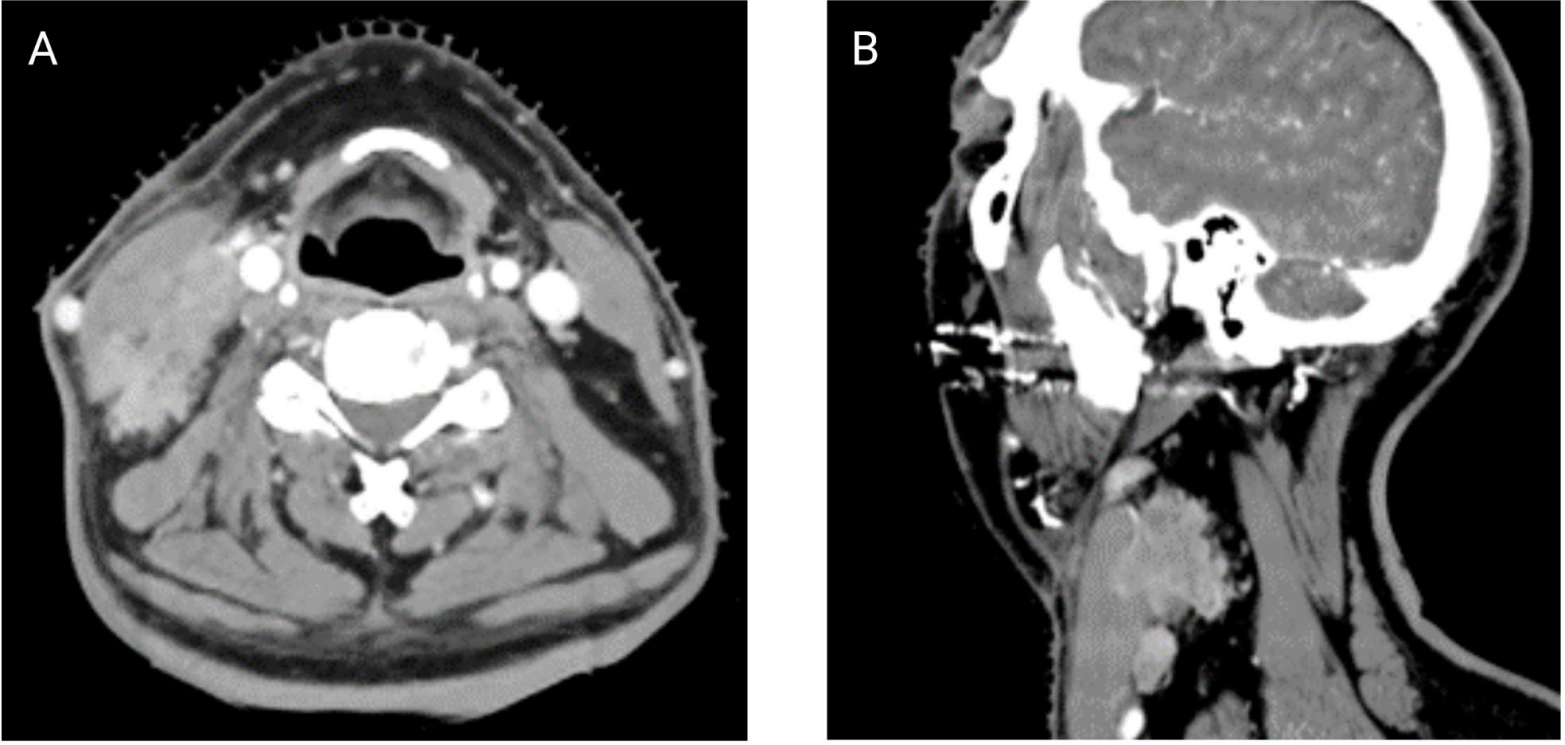
Contrasted axial **(A)** and sagittal **(B)** CT scans of a patient with clear ENE (Images kindly provided by Dr. Santiago Medrano).

**Figure 3:**
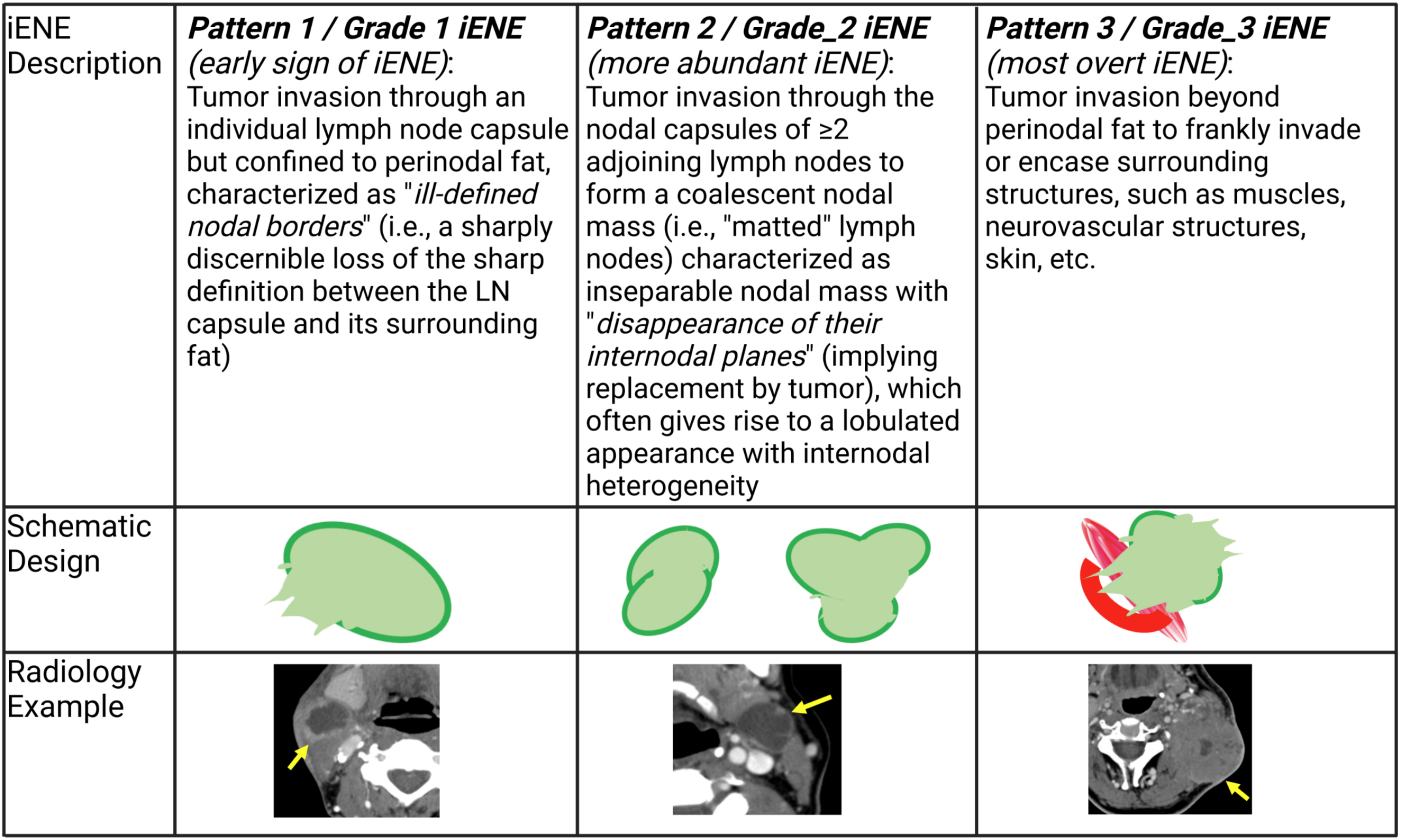
Pattern/Grade of radiologically imaged ENE – depicting the extent of image-identified ENE (iENE**)** based on Hoebers et al. (28) and Chin et al. (29) (Images kindly provided by Dr. Eugene Yu).

**Figure 4:**
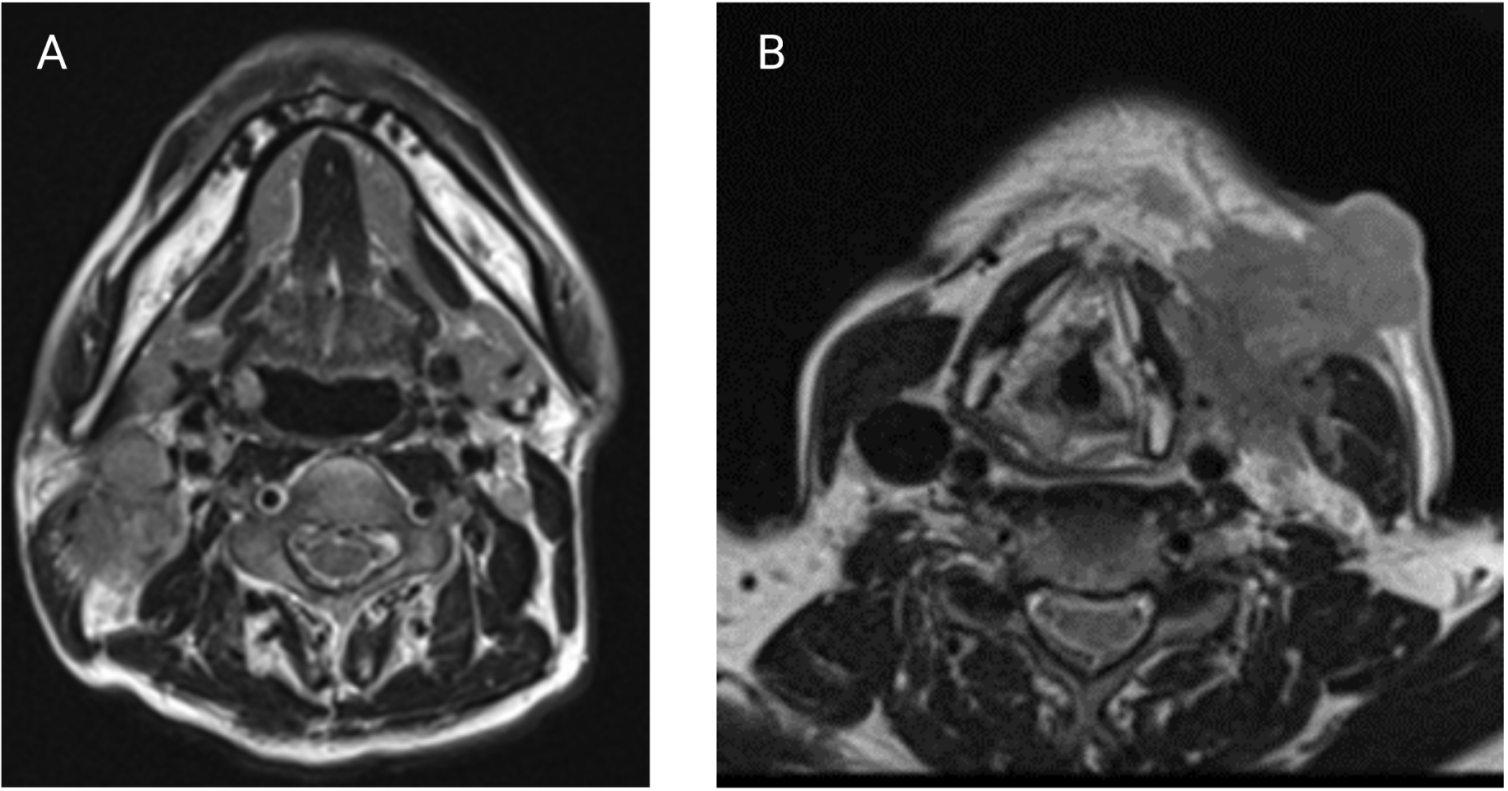
**(A)** Axial T2-weighted MRI showing a coalescent right level II nodal mass suspicious for iENE. **(B)** Axial T2-weighted MRI with a large L level III nodal mass with clear invasion of the sternocleidomastoid and extension into overlying subcutaneous fat and skin. (Images kindly provided by Dr. Eugene Yu).

ENE can also be visualized on pre-treatment morphologic imaging such as computed tomographic scans (CT) (18), magnetic resonance imaging (MRI) (19), ultrasound scans (US) (20) and positron emission tomography-computed tomographic scans (PET-CT) (21). Whilst the role of CT and MRI is well-established in the diagnosis of iENE, the value of PET-CT or US is questioned by many (22). There are several other issues that remain unresolved in defining or diagnosing iENE. Firstly, there is no clear consensus on the terminology, definitions or diagnostic criteria. iENE is often defined as an involved LN on imaging with an unequivocally ill-defined nodal border (**Figure 2**), i.e., clearly discernible loss of the sharp plane between LN capsule and surrounding fat (23–26). Faraji et al. (27) performed a retrospective analysis of preoperative CT images of patients with HPV-associated HNSCC and concluded that irregular nodal margins and absence of perinodal fat plane were the most specific and sensitive features for iENE. The terms “conglomerate”, “matted” and “coalescent” have all been used to describe radiographically poorly delineated aggregates of two or more LNs, where iENE occurs between abutting nodes with loss of the intervening nodal planes (**Figure 3**, pattern 2; **Figure 4A**) (5, 26, 30). However, there are limitations and some differences between these terms, even though many might use and interpret them interchangeably (31, 32). Another area of uncertainty in iENE reporting is the presence of central nodal necrosis. Some studies have recognized central nodal necrosis as a significant predictor of ENE, but others have dismissed it as an intranodal characteristic rather than a feature of ENE (33–35). It is possible that nodal necrosis is only an association with ENE since both might indicate an aggressive tumor phenotype).

**Figure 5:**
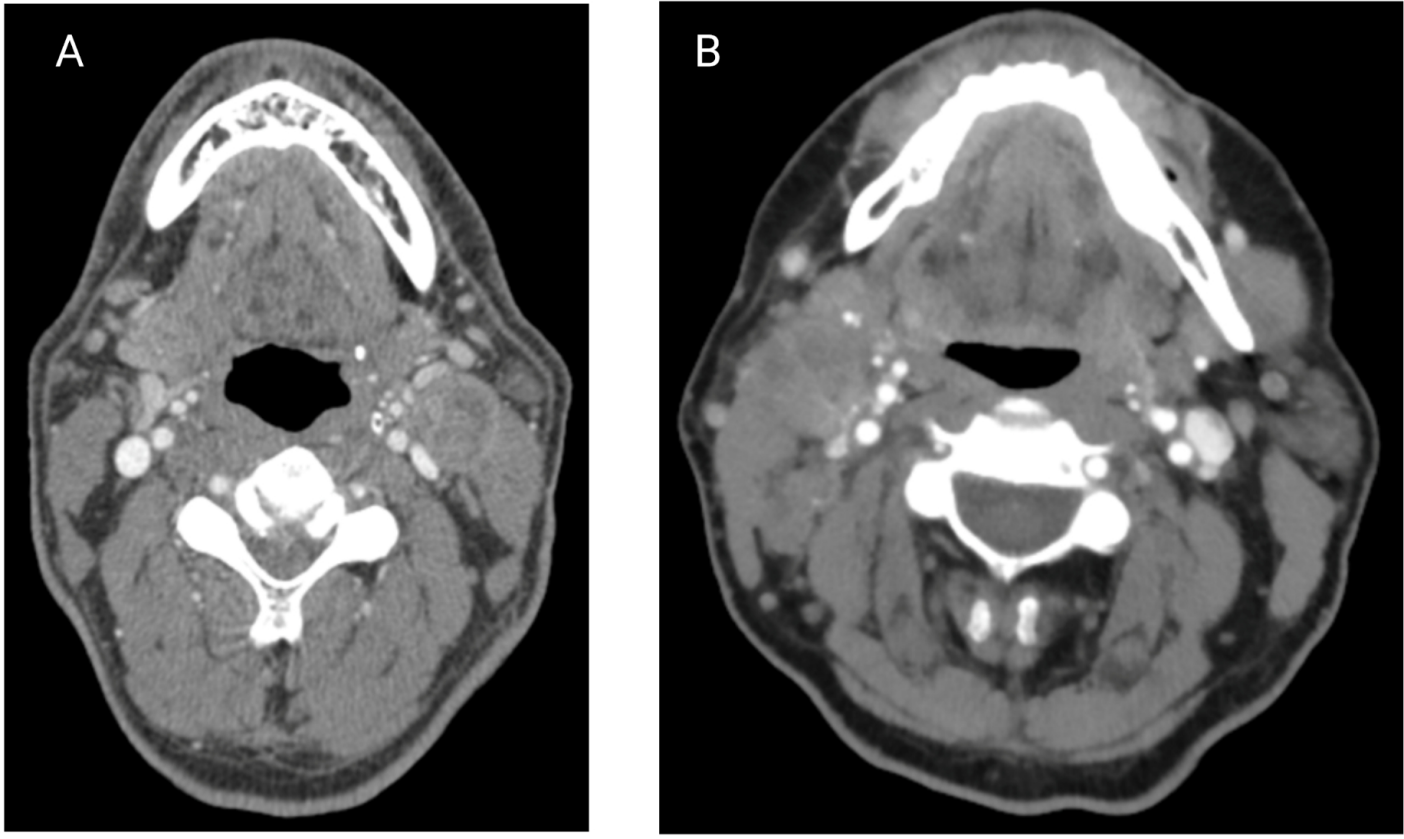
**(A)** Contrasted axial CT scan of a patient with a L level II node suspicious for iENE, which was later confirmed pathologically. **(B)** Contrasted axial CT scan of a patient with a R level II node suspicious for iENE, but ENE was not found on pathology. (Images kindly provided by Dr. James Bates).

Unfortunately, the use of imaging for ENE diagnosis prior to treatment is complicated by reports of low sensitivity, and poor negative predictive value (**Figure 5**). Studies show that iENE demonstrates a sensitivity of 60-80% and a specificity of 72-96% to predict pENE (17, 36, 37). Maxwell et al. (38) assessed CT scans from 65 surgically treated HNSCC patients, with two radiologists scoring the likelihood of iENE using a 5-point scale. That method demonstrated high inter-rater variability and poor performance, with an area under the curve (AUC) of the receiver operating characteristic ranged from 0.65–0.69 (38). This has stimulated research into methods to improve iENE reporting, and as a result, various imaging features have been combined into grading or classification systems, **Table 1**. Chin et al. (29, 39), Hoebers et al. (28) and Lu et al. (31) have all classified iENE into three patterns (**Figure 3**; **Table 1**), while Ai et al. (40) categorized the presence of iENE into just two grades. All four systems identified grade 1 as iENE limited to perinodal fat only. Moreover, both Hoebers’ and Lu’s systems aligned well for grade 2 (coalescent or matted nodes) and grade 3 (ENE into adjacent structures like muscles, nerves, skin, etc.). Ai et al. omitted coalescent or matted nodes as feature from their classification (40).

**Table 1:** The classification systems published for iENE reporting.

**Authors**	**Year of publication**	**Number of tiers**	**Classification system details**
Hoebers et al. (28)	2022	4 tiers	ENE Grade 1: Tumor invasion through the nodal capsule of an individual LN with unambiguous ill-defined nodal border(s), but confined to perinodal fat ENE Grade 2: Tumor invasion through two or more inseparable adjoining nodes exhibiting unambiguous effacement of any component of their internodal plane(s) (implying replacement by tumor, that is, extranodal extension), which invariably produces a lobulated appearing nodal mass ENE Grade 3: Tumor invading beyond perinodal fat to overtly invade or encase adjacent structures, for example, skin, muscle, neurovascular structures, etc. ENE negative: All other cases with none of these radiological features of iENE, or cases which are equivocal or uncertain
Chin et al. (29)	2022	3 tiers	ENE Grade 1: Radiological sign of tumor breaching capsule of an individual node with unambiguously ill-defined nodal border(s) but confined to perinodal fat ENE Grade 2: Radiological sign of tumor invasion through two or more inseparable adjoining nodes exhibiting unambiguous effacement of any component of their internodal plane(s), invariably resulting in a lobulated appearing nodal mass ENE Grade 3: Tumor invading beyond perinodal fat to overtly invade or encase adjacent structures, e.g., skin, muscle, and neurovascular structures
Chin et al. (39)	2021	3 tiers	ENE Grade 1: Tumor invading individual/separate node(s) capsule(s) but confined to perinodal fat, characterized by clearly discernible loss of the sharp definition between the nodal capsule and its surrounding fat, or irregular nodal borders in the individual nodes. ENE Grade 2: Tumor invading through two or more adjacent nodes which merge to form a coalescent nodal mass characterized by partial or complete loss of the intervening planes. ENE Grade 3: Tumor extending beyond perinodal fat to overtly invade or encase adjacent structures, such as muscles, neurovascular structures, parotid, or skin. Encasement was defined as juxtaposed tumor encircling an anatomical structure by ≥270°.
Lu et al. (31)	2019	4 tiers	ENE Grade 1: overt LN with infiltration into surrounding fat plane only ENE Grade 2: coalescent LNs (comprised of ≥2 LNs) with clear evidence of iENE) ENE Grade 3: tumor invading beyond LN capsule into adjacent structures (i.e., muscles, nerves, parotid glands, etc.) ENE negative: All other cases with none of these radiological features of iENE or those that are Equivocal/uncertain cases
Ai et al. (40)	2019	3 tiers	Grade 0: node without ENE Grade 1: node with ENE infiltrating surrounding fat Grade 2: node with ENE infiltrating adjacent muscle and/or skin and/or salivary glands

ENE, extranodal extension; iENE, ENE on imaging.

### 2.3 Clinical ENE

Clinical ENE (cENE) was defined by the AJCC 8^th^ edition of the TNM staging manual as: 1) unambiguous evidence of gross ENE on clinical examination, i.e. invasion of skin, infiltration of musculature or dense tethering to adjacent structures or cranial nerve, brachial plexus, sympathetic trunk or phrenic nerve invasion with dysfunction, and 2) strong radiographic evidence of ENE to support the clinical examination (9) (**Figure 4B**). Clinical ENE will typically correlate with grade 3 in the Hoebers and Lu classification systems (28, 31, 41). As such, this designation is reserved for only the most obvious, and relatively uncommon, cases of ENE.

## 3 Prognostic impact of ENE

### 3.1 Prognostic impact of pENE

pENE typically indicates a poorer prognosis (42, 43). While some of the older studies concluded that there was no relationship between pENE and survival (44, 45), these studies exhibited significant weakness due to insufficient statistical power, heterogeneity in adjuvant treatment strategies, and variable pathological interpretation of pENE (46, 47). More recent studies examining the prognostic importance of pENE in HNSCC have reported up to 50% lower relative overall and disease-specific survival (DSS) for patients with pENE (43, 48–55). Several publications (43, 46, 52, 56) have demonstrated that pENE is a poor prognostic indicator for distant metastasis (DM) (pooled OR 2.18, 95% CI 1.23–3.87), and loco-regional recurrence (LRR) (pooled OR 1.33, 95% CI 0.86–2.07). Other studies have concluded that pENE is a better predictor of OS than either resection margins (50, 57, 58) or TNM staging (43, 57, 58).

Based on its prognostic importance, pENE is considered an indication for intensification in treatment protocols for HNSCC patients. A pooled subset analysis of two landmark trials [EORTC 22931 (7) and RTOG 9501 (8)] cemented the paradigm of treatment intensification for patients with pENE (6). The addition of concomitant high-dose cisplatin to RT in patients with pENE and/or positive margins reduced the risk of LRR and death by 42% and 30% respectively compared to adjuvant RT alone (6). Although these trials did not analyze pENE and positive margins separately, they still support the role of intensified treatment in pENE cases as over 50% of enrolled patients had pENE (5, 6). Importantly, this adjuvant therapy for cases with pENE attenuates the reported negative impacts of pENE on prognosis (5, 59). It should be noted however that these studies consisted mainly of non-oropharyngeal cancer and so recruited cases were highly likely to be mainly HPV-negative.

### 3.2 Prognostic impact of the extent of ENE

The significance of the extent of pENE remains unclear. This controversy potentially stems from the lack of a universally accepted pENE definition and is further confounded by variations in sample processing and interpretation (5, 47, 49). While macroscopic pENE consistently indicates poor prognosis in HNSCC (60–62), the prognostic significance of microscopic pENE has not been widely proven (49, 50, 60, 63). Carter et al. (63) and Brasilino de Carvalho (60) found that macroscopic pENE increased the risk of recurrence (RR 3.5, 95% CI 1.7–7.0), and worsened recurrence-free survival (RFS), but they found that microscopic pENE had no impact (RR 1.3, 95% CI 0.6–3.0). Similarly, Clark et al. (62) studied a mixed cohort of HNSCC with advanced nodal stage and reported that while microscopic pENE had a similar risk of regional recurrence as no pENE, those with macroscopic pENE fared significantly worse (RR 4.3; 95% CI 1.87–9.89). However, the authors noted that patients with microscopic pENE had intermediate DSS outcomes between those with no pENE and those with macroscopic pENE, but the authors did not report actual values (62). Moreover, Jose et al. (49) analysed survival outcomes in a mixed cohort of HNSCC patients (71% had laryngeal and hypopharyngeal cancers), and demonstrated no statistical difference in RFS between microscopic and macroscopic pENE. It is apparent that there is differential impact of pENE by its extent. However, optimal cutoff of pENE extent remains debatable. The 8^th^ edition AJCC/UICC (TNM8) staging manual did not include a cut-off for the extent of pENE in its definition, but they recommended documenting pENE grade as minor (≤2mm) or major (>2mm) ENE (64). Wreesmann et al. (65) and Mamic et al. (13) used ROC curve analysis to define discriminatory thresholds of 1.7 and 1.9 mm respectively in patients with oral cavity cancers, supporting the AJCC recommendation of 2 mm cutoff threshold. de Almeida et al. also showed prognostic difference in minor vs major pENE using a 2 mm cutoff (66). Similarly, Kwon et al. (11) and Arun et al. (16) found that empirically defined pENE extension thresholds of 2 mm (in a mixed cohort of HNSCC) and 5 mm (in oral cavity cancers) respectively, were able to produce significant survival differences (HR 3.8, 95% CI 2.0-7.2, and HR 95% CI). However, other studies did not support the arbitrary 2 mm extension threshold (15, 16). At the least, future efforts to better define and standardize pENE criteria would be well-served by agreeing upon a consistent terminology, i.e., major/minor or macroscopic/microscopic. Since ENE is most often diagnosed by a pathologist using a microscope and very rarely by the naked eye, even when greater than 2mm, we suggest the use of the terms major/minor as opposed to microscopic/macroscopic.

### 3.3 Prognostic impact of pENE in HPV-associated oropharyngeal squamous cell carcinomas

The impact of pENE on prognosis of HPV-associated OPSCC is widely questioned (67–69). Multiple retrospective single-centre studies (67, 69–79), two multi-centric studies (80, 81), one small national cancer database (NCDB) study (82), and two systematic reviews (46, 59) failed to demonstrate a negative prognostic impact for pENE in HPV-associated OPSCC treated with surgery and post-operative adjuvant therapy and suggest that the addition of chemotherapy to adjuvant radiation may not be necessary in such cases. Consequently the AJCC/UICC staging system excluded pENE for those patients (5, 64, 83) However, it must be noted that most of these studies were small and arguably inadequately powered to detect statistical significance for pENE in HPV-associated OPSCC, especially in this group of patients with significantly better survival and fewer events (5, 83, 84). Furthermore, the use of adjuvant chemoradiotherapy may have mitigated the negative prognostic impact of the pENE.

More recently, increasing evidence has suggested that pENE (especially >1 mm) in HPV-associated OPSCC does indeed negatively impact survival and regional control (83–90). Multivariate analysis of 92 p16-positive patients found pENE was an independent risk factor for overall survival (OS) and disease progression (87). Several other retrospective analyses of patients with HPV-associated OPSCC show that pENE is associated with worse survival, albeit with moderate effect size (5-11%) (84, 88–90). Importantly, a recent systematic review by Benchitrit et al. (83) pooling 1349 patients from 6 studies, concluded that pENE is associated with a relative reduction in OS of 89% (HR 1.89, 95% CI 1.15-3.13) in HPV-associated OPSCC. Moreover, the phase II ECOG 3311 trial (91) demonstrated that arm D, in which 88% of low risk HPV-associated OPSCC patients had pENE >1 mm, showed significantly poorer 2-year progression-free survival (PFS) of 90.7% (90% CI, 86.2 to 95.4) compared to arm A patients (2 year PFS= 96.9% (90% CI, 91.9 to 100%), who had no pENE, and arm B and C patients who had <1 mm pENE (2year PFS 94.9% (90% CI, 91.3 to 98.6]) and 96.0% (90% CI, 92.8 to 99.3) respectively. These differences in OS are further underscored by the fact that arm D patients received significantly more intensive tri-modal therapy (surgery, 66Gy of RT and cisplatin), compared to arm A patients (surgery only), and arm B and C (surgery and RT only at 50 or 60 Gy) (91).

### 3.4 Prognostic impact of iENE

Since the evidence on pENE only applies to surgically treated patients, there is great interest in the prognostic value of iENE, which could be applied to a wider cohort of HNSCC patients (5, 59, 83). In a systematic review, Benchetrit et al. (83) pooled iENE data from 1468 patients with HPV-associated OPSCC and demonstrated that iENE led to worse OS (HR 2.64, 95% CI 1.46-4.78), with a greater contribution by increased risk of distant failure (HR 3.83, 95% CI 1.88-7.80) than locoregional failure (HR, 2.03, 95% CI 0.86-4.79). A similar trend is also seen in the nasopharyngeal (30, 31, 39, 92) and HPV-negative HNSCC (41) literature: one large systematic review (93) that pooled data from 7532 patients with nasopharyngeal cancer found that iENE was associated with worse OS (HR 1.85-2.62) and distant metastasis-free survival (HR 2.07 -3.14). iENE has also been shown to be an independent factor associated with worse survival and distant control in patients with HPV-negative tumours in the oropharynx (41), oral cavity (23) and in mixed cohorts of HNSCC (94–96). Together, this literature points to a higher prognostic impact for iENE than pENE, possibly indicating that unequivocal iENE correlates with more advanced pENE (23, 83).

## 4 Management of ENE in head and neck cancer

To date, identification of pENE in HNSCC is critical in determining optimal treatment. Moreover, the identification of pENE also dictates the radiation dose. Peters et al. (97) conducted a prospective randomized trial to evaluate the optimal dose of radiotherapy in patients with locally advanced HNSCC. In this study, lower risk patients were randomized to 57.6 Gy vs 63 Gy, and those who were higher risk (typically pENE or positive margins) were randomized to 63 Gy vs 68.4 Gy. The study demonstrated that patients who had pENE had better regional control with doses ≥63 Gy (97). These results were further validated in a prospective clinical trial in 288 patients with locally advanced HNSCC (98). High risk patients with pENE or ≥2 risk factors received a higher dose, 63 Gy over either 5 or 7 weeks and showed that altered (accelerated) fractionation trended towards improved loco-regional control and OS.

The identification of ENE is also important in HPV-associated OPSCC, a disease setting typically associated with a favorable prognosis (99, 100). The pre-treatment detection of ENE in those patients plays a crucial role in identifying patients who may benefit from treatment de-escalation or intensified treatment approaches. For example, in ECOG 3311, even though patients with matted nodes on radiology were excluded from participating, nearly 30% of recruited patients had pENE mandating an escalation of adjuvant treatment, mainly for pENE >1mm (91). In the ORATOR trial, which compared primary RT versus surgery for patients with early OPSCC (mostly p16-positive) and no signs of iENE, nearly 24% of the surgery group had high risk features (positive margins or/and pENE) and ended up receiving tri-modal therapy (101). A recent analysis of a large national cancer database demonstrated pENE in up to 28% of patients who underwent transoral robotic surgery for HPV-associated OPSCC (102). These patients may not have required tri-modal therapy, if ENE could have been reliably identified before surgery, and definitive chemoradiotherapy recommended instead.

Based on conflicting evidence regarding the prognostic significance of pENE in HPV-associated OPSCC, there have been some studies exploring the de-escalation of treatment in surgically treated patients. The AVOID study attempted to omit chemotherapy for surgically treated HPV-associated OPSCC patients with no pENE and showed an excellent 2-year PFS rate (92.1%, 95% CI 80.2%-97.0%) (103). However, if one extrapolates the results of the ECOG 3311 one can surmise that in patients with pENE <1mm, dose reduction may be feasible, albeit that did not result in major improvements in patient reported functional outcomes (91). However, patients with pENE >1mm appear to have poorer outcomes than those without, despite receiving significantly more intensive treatment with cisplatin and higher doses of RT. In summary, although there may be a group of patients with HPV-associated OPSCC with low burden of ENE (possibly those with less than 2mm of ENE) who may not actually benefit from chemotherapy, that subgroup has not been adequately identified and therefore the treatment of such patients with adjuvant RT alone is not appropriate outside of a clinical trial.

## 5 Gaps in knowledge and future considerations

### 5.1 pENE definitions and terminology

The recent changes to the AJCC/UICC TNM system (64) were well received by the head and neck oncologic community worldwide (104), but the criteria for “unambiguous”, clinically-overt ENE (105) remain vague. Moreover, there is significant uncertainty regarding the diagnostic criteria of pENE, leading to heterogeneity when making a diagnosis. This may be contributing to the conflicting evidence commonly encountered in pENE research. There is still no consensus among pathologists on a preferred terminology for pENE, and a constellation of terms like extranodal extension, extracapsular spread, or extranodal spread are used interchangeably. Furthermore, while most pathologists will agree that extension of tumor cells in the perinodal fat and soft tissue is diagnostic for pENE, there is still significant uncertainty around determining pENE in challenging cases with matted nodes, nodal hilar involvement or in cases with direct extension of primary tumor into a node. Moreover, there is still no agreement between pathologists whether HPV status should be taken into consideration when interpreting pENE features. These challenging issues are best addressed first by consensus, to standardize the definitions, terminologies and synoptic reporting used for pENE, especially for microscopic versus macroscopic, in both HPV-associated and HPV-negative HNSCC. The pathology community should also come to agreement on a standardized lymph node processing and sampling methodology for pENE (5, 10). This may help facilitate much needed research on the prognostic power of pENE in both HPV-associated and -negative HNSCC.

### 5.2 iENE definitions and terminology

The importance of a common language for defining iENE at the time of diagnosis has been poorly addressed so far, and the lack of standardized definitions and nomenclature has contributed to conflicting evidence, both in clinical trials and real-world data (5). However, a rational roadmap to develop a standardized nomenclature for iENE faces some challenges. Widespread agreement in the radiology community regarding the diagnostic criteria for ENE on imaging is still lacking. Features like nodal size, central nodal necrosis and capsular thickening are still being debated as criteria for iENE. Moreover, there is still no consensus regarding interpreting findings like matted/coalescent nodes, or the impact of HPV status on iENE features. Furthermore, there is still no conclusive evidence regarding the best imaging modality for iENE identification. There are several published classification systems for iENE in head and neck cancer as shown in **Table 1**, but none of these systems have been widely adopted in routine clinical practice. Research into assessing, improving and validating these systems is needed, to enable wide adoption into clinical practice and there is a need for better appreciation of the impact of ENE, at least in its worst form, on the outcome of patients rather than disregarding it.

Thus, there is a pressing need for standardized diagnostic criteria for iENE, which could improve reproducibility and facilitate research and widespread clinical implementation. In our view, an international consensus process aiming to standardize the iENE criteria and address these gaps in the literature on iENE is needed.

### 5.3 Prognostic impact of iENE and pENE in HPV-associated and HPV-negative HNSCC

Robust and large-scale studies are needed to quantify the prognostic impact of pENE and iENE in HPV- associated and HPV-negative HNSCC. Such studies need to be adequately powered to definitively address the prognostic significance of the different grades of pENE, and to validate the commonly used 2 mm threshold, especially in HPV-associated tumors. This could then be integrated into the TNM system, de-escalation trials, and everyday practice.

### 5.4 Artificial intelligence for iENE

Recent advances in artificial intelligence (AI) may hold promise for use in the diagnosis of iENE and outcome prediction. Kann et al. (106) trained a 3-dimensional convolutional neural network using 2875 CT-segmented LN samples, correlated with pathology samples to act as a ground truth. They demonstrated an improvement in the AUC to 0.91 with a sensitivity of 88% (false negative rate: 12%), and specificity of 85% (false positive rate: 15%) (106). They later validated this approach using two external cohorts, consisting of a total of 200 LNs (107). The algorithm achieved an AUC of 0.84 (83.1% accuracy) and 0.90 (88.6% accuracy) in the two cohorts, outperforming two independent radiologists’ AUCs of 0.70 and 0.71 in the first cohort, and 0.60 and 0.82 in the second cohort respectively. The diagnostic accuracy and inter-rater variability of both radiologists improved when they were supported with deep learning assistance. Ariji et al. (108) also developed a deep learning algorithm and compared performance to radiologists. Once again, the deep learning system achieved high accuracy (84.0%) for diagnosing iENE, using a set of AI-determined features. In comparison, the radiologists’ accuracies based on a set of radiological criteria - minor axis ≥ 11 mm, central necrosis, and irregular borders- were 55.7%, 51.1% and 62.6% respectively (108). These efforts are still in early stages of development and will need to undergo wider external validation before routine implementation in clinical practice.

### 5.5 Biomarker discoveries for iENE

In the last decade, advances in biomarker technologies have led to multiple discoveries in HNSCC diagnosis and prognosis. There are currently several promising molecular biomarkers that could potentially be used for predicting pENE before commencement of treatment. However, these are still in early stages of development, with a high rate of false discoveries (46, 109). External validation in larger cohorts, and in some cases, better biomarkers are needed to confirm these associations and their clinical impact before being incorporated into clinical treatment paradigms.

## 6 Conclusion

Extranodal extension is associated with aggressive cancer behavior and poor prognosis. There are challenges in accurately identifying and classifying ENE, both on histopathologic examination and on pre-treatment imaging. Although earlier single institutional studies (72, 76) suggested lack of impact of pENE on HPV-associated OPSCC, likely due to selection bias and small sample size, more recent large studies indicate pENE is prognostic in this disease (83, 88, 89). iENE also has a negative prognostic impact, particularly on distant control. One of the major challenges is how to reduce the risk of distant metastasis in ENE+ patients (89, 110). International consensus is needed on definitions, terminology, and diagnostic criteria for both pENE and iENE in HNSCC. Moreover, large-scale studies are necessary to determine their prognostic impact in HPV-associated and HPV-negative cases.

## Author contributions

CH: Conceptualization, Writing – original draft, Writing – review & editing. AA-F: Conceptualization, Writing – original draft, Writing – review and editing. DM: Writing – original draft, Writing – review and editing. LM: Conceptualization, Writing – original draft, Writing – review and editing. SB: Conceptualization, Writing – original draft, Writing – review and editing. JB: Conceptualization, Writing – original draft, Writing – review and editing. AL: Conceptualization, Writing – original draft, Writing – review and editing. PB: Conceptualization, Writing – original draft, Writing – review and editing. PS: Conceptualization, Writing – original draft, Writing – review and editing. PN: Conceptualization, Writing – original draft, Writing – review and editing. SH: Writing – review and editing. WL: Writing – review and editing. BO: Writing – review and editing. HM: Conceptualization, Writing – original draft, Writing – review and editing.
